# Interventions about physical activity and diet and their impact on adolescent and young adult cancer survivors: a Prisma systematic review

**DOI:** 10.1007/s00520-024-08516-0

**Published:** 2024-05-13

**Authors:** M. Vasilopoulou, Z. Asimakopoulou, J. Velissari, A. Vicha, M. Rizogianni, S. Pusa, S. Stöven, S. Ficarra, Α. Bianco, D. Jiménez-Pavón, G. Ponce Gonzalez Jesus, C. Tavares Paula, B. Gomes, D. Bayer, S. Silva, C. Nogueira, S. Papakonstantinou, K. Musa, F. Glorioso, A. Vantarakis

**Affiliations:** 1 Department of Public Health, Medical School, University of Patras, Patras, Greece; 2 Oncology Unit, University General Hospital of Patras, Patras, Greece; 3 Department of Nursing, Umeå University, Campus Örnsköldsvik, Box 843, S 891 18 Örnsköldsvik, Sweden; 4 Sport and Exercise Sciences Research Unit, Department of Psychology, Educational Science and Human Movement, University of Palermo, Via Giovanni Pascoli 6, 90144 Palermo, Italy; 5 Department of Physical Education, Faculty of Education Sciences, University of Cádiz, Puerto Real, Spain; 6 Biomedical Research and Innovation Institute of Cádiz, University of Cádiz, Cádiz, Spain; 7 CIBER of Frailty and Healthy Aging (CIBERFES), Instituto de Salud Carlos III, Madrid, Spain; 8 Faculty of Sport Sciences and Physical Education and *CIDAF (Uid/Dtp/042143/2020), University of Coimbra, Coimbra, Portugal; 9 Department of Health Consulting, Research and Science, Outdoor Against Cancer, Munich, Germany; 10 Psycho-Oncology Unit, Portuguese Cancer League–Centre Branch, Lisbon, Portugal; 11 Innovation & Development Unit, Coimbra Hospital and University Centre (CHUC), Coimbra, Portugal; 12 European CBRNE Center, Umeå University, KBC-Huset, Linnaeus Väg 6, 90187 Umeå, Sweden; 13 Creative Thinking Development-CRE.THI.DEV, Rafina, Greece; 14 CEIPES ETS, Palermo, Italy; 15 Lega Italiana Per La Lotta Contro I Tumori (LILT Palermo), Palermo, Italy

**Keywords:** Adolescent young adult cancer survivors, Diet, Physical activity, Intervention

## Abstract

**Purpose:**

Over the past few decades, the incidence of cancer among adolescents and young adults (AYA) has been increasing. The impact of behaviors, such as physical activity (PA) and nutrition, on disease progression, prognosis, and overall health and quality of life for AYA cancer survivors is of significant importance. This systematic review aims to evaluate the effectiveness of PA and diet interventions for AYA cancer survivors and to critically evaluate existing literature, gaps, and limitations.

**Methods:**

A search of literature was conducted in PubMed, Science Direct, Scopus, and Google Scholar following the PRISMA guidelines. Twenty-two studies were included from online databases from 2012 to 2022, 13 of which were randomized controlled trials.

**Results:**

Most interventions were related to PA, with only four studies including nutrition or Diet interventions. The interventions were generally feasible and acceptable to AYA cancer survivors, and digitally based PA interventions were commonly used. PA interventions mainly comprised aerobic and resistance training and were individualized. Overall, this review found various PA and diet interventions for AYA cancer survivors that were feasible and well-accepted, but gaps in knowledge and design still exist.

**Conclusions:**

This systematic review underscores the importance of conducting more research on diet interventions for YCS.

**Prospero registration:**

https://www.crd.york.ac.uk/prospero/#aboutregpage

**Supplementary Information:**

The online version contains supplementary material available at 10.1007/s00520-024-08516-0.

## Introduction

The incidence of cancer in the adolescent and young adult (AYA) age group has been on an upward trend in recent decades (National Cancer Institute (NCI), 2023a). AΥΑs cancer survivors are recognized as a distinct subgroup among oncology patients and are defined as individuals with cancer diagnosis between the ages of 15 and 39 years [1],NCI, 2023b), encompassing mid- and late adolescence (15–17 years), emerging adulthood (18–25 years), and young adulthood (25–39 years) [2]. AYAs cancer survivors also face serious medical, psychosocial, and behavioral late effects associated with cancer and its treatment, such as risk of cardiovascular disease, diabetes, cancer recurrence, fatigue, obesity, weight gain due to treatment, and psychological distress [3–7]. Behavioral factors, such as physical activity and nutrition, are strongly related to disease progression and prognosis [8, 9] late adverse effects of treatment, overall health, and quality of life of AYAs (DeNysschen, Panek – Shirley & Zimmerman, 2021 [10],).

The World Health Organization (WHO) defines physical activity as “any bodily movement produced by skeletal muscles that requires energy expenditure” and is considered an effective intervention and health promotion tool for AYAs cancer survivors (World Health Organization: WHO, 2022). The protective role of PA in health is well established and is associated with numerous health benefits, such as health-related fitness and quality of life [11, 12], disease-free survival rates, cardiovascular fitness, wellbeing, and decreased all-cause cancer mortality [9, 12–14]. However, PA often is followed by physical inactivity [12, 15], with research findings suggesting that around 60% of young adults with cancer do not meet the recommended PA guidelines (150 min of moderate to vigorous PA per week (Cambell et al., 2019,[16]. Furthermore, considering their specific clinical, developmental, physical, and psychosocial needs, adolescent and young adult cancer survivors experience various physical and psychosocial challenges [5] such as fatigue [17, 18], significant weight loss or gain, and chronic conditions like diabetes and obesity [19] alongside psychological and social distress [20], and finally a negative body image [21]. These parameters are negatively associated with the adoption, re-engagement, and commitment to an active lifestyle through PA and exercise after cancer treatment [12, 22] which is particularly important given that more than 80% of AYAs will survive more than 5 years after the onset of their cancer diagnosis (Close et al., 2019 [14],).

Diet is among the behavioral immunogens along with PA [23] and an important health behavior for the entire trajectory of cancer survivorship [24]. The side effects and symptoms of cancer treatment related to a survivor’s diet such as changes in sense of taste, intestinal disorders, and difficulty in chewing and swallowing still occur [25]. Metabolic disruptions are a common side effect of cancer treatment for AYA, while a healthy eating pattern may reduce the likelihood and risk of metabolic comorbidities [26, 27]. There is a strong correlation between diet and cancer mortality. Research indicates that many adolescents and young adults who have survived cancer have inadequate dietary habits, with approximately 80% of them not consuming enough fruits and vegetables, still, despite their low intake, their consumption of fruits and vegetables is similar to that of the general population [28]. A diet rich in fiber, fruits, and vegetables, like the Mediterranean diet, has been shown to reduce cancer mortality [29, 30], the risk of cardiovascular disease, diabetes, and obesity [7, 31]. Studies focusing on interventions that seek to improve nutrition education have found that making healthy nutrition choices and understanding how to read and to identify saturated fats can be enhanced after a Diet intervention program targeting AYA cancer survivors but with no apparent significant difference in dietary intake [11]. Providing nutrition education to young cancer survivors enhances their understanding of dietary choices, yet the adoption of healthier eating habits may vary depending on factors such as age, level of independence, and the intensity of educational sessions [11].

There are guidelines for cancer survivors and for AYA cancer survivors focusing on weight management, PA, and dietary patterns which highlight the potential impact on the risk of recurrence and overall survival after a cancer diagnosis [7]. Considering that in the last decade, there has been an increase in cases of adolescents and young adults with cancer. Hence, a systematic approach aiming to investigate effective interventions that address both physical activity and nutrition among AYA cancer survivors could enhance the existing knowledge of health behaviors in this population and frame their needs holistically.

The purpose of this systematic review was to identify interventions between 2012 and 2022 regarding physical activity and diet and their effectiveness for adolescent and young adult cancer survivors. To the best of our knowledge, this represents the first extensive review covering both physical activity and diet interventions for adult and young adult cancer survivors.

## Materials and methods

This systematic review was conducted in accordance with the guidelines outlined in the PRISMA (Preferred Reporting Items for Systematic Reviews and Meta-Analyses) recommendations to ensure that best practice in this area is followed [32]. The current review protocol was registered in the PROSPERO database on record (Reg: CRD42024531628).

### Search strategy

A systematic literature search was conducted between September 2022 and February 2023 using four databases: PubMed, Science Direct, Scopus, and Google Scholar. Original studies examining the effects of physical activity and diet interventions in adolescent and young adult (AYA) cancer survivors were screened. The search consisted of the medical subject headings (MeSH) and text words (a) concerning target group and condition, “adolescent,” “young adult,” “young adult cancer,” “young adult cancer survivors,” “AYA cancer survivors,” “cancer,” “survivors,” “survivorship”; (b) concerning intervention, “health behaviors,” “lifestyle,” “diet,” “nutrition,” “physical activity,” “exercise,” and “intervention.” One example of the full electronic database search for one database with all additional filters can be found in the Electronic supplementary material.

A secondary search was conducted by manually reviewing the reference sections of relevant studies including only those with available full texts. The authors were contacted for unavailable full texts; however, studies were excluded if authors were unresponsive or unable to provide the complete text. The search queries were set to include the above terms in the article title.

### Eligibility criteria

Upon completion of the title screening, a set of inclusion and exclusion criteria were applied during the abstract screening as well as in all full-text sections.

Papers were considered eligible for review if they met the following criteria: (1) they were published in a peer-reviewed journal, (2) they focused on survivors of any type of cancer who were diagnosed and treated during adolescence (15–18 years) or young adulthood (19–39 years) and included an intervention that targeted physical activity and/or diet, (3) they were written in English, (4) they were published within the last decade (2012–2022), as the aim of the review is to present the most recent data in this field, and (5) they did not necessarily need to include a healthy control group. In contrast, papers were ineligible if they employed a summative measure of multiple health behaviors without providing individual behavior results, presented solely qualitative data, involved patients who were still receiving treatment, or were literature reviews and case studies. Studies were additionally excluded if most participants did not fall within the selected age range or if they were childhood and/or older cancer survivors. Articles that did not provide a clear number of cases or covered only a partial period that did not align with our systematic review as well as expert opinions, hand-searched sources, conference proceedings, articles, and letters to the editor were excluded.

### Study selection

All results obtained from the database search were compiled in EndNote (Clarivate Analytics, PA, USA). Duplicate articles were first removed from the software and then manually checked by the two independent reviewers (MV and ZA). In the initial phase of the study, the two independent reviewers assessed the titles, abstracts, and keywords to determine whether the studies met the criteria for inclusion. All abstracts that did not meet the inclusion criteria were rejected. Any study that was considered relevant or provided incomplete information (as reviewed by the abstracts) was included in the articles for full-text review. A third reviewer (AV) followed the same procedure and assessed study eligibility using the same standardized approach. After the third reviewer independently agreed with the results, any differences were discussed and resolved among all authors, resulting in a final consensus.

### Data extraction and quality assessment process and items

Two authors (MV and ZA) independently reviewed 123 full-text articles screened for eligibility and then extracted data for the included studies, including author, year of publication, country of study, sample size, age of participants, and type of cancer. Subsequently, to achieve our main objective, i.e., to compile the PA and diet interventions in AYA cancer survivors and their qualitative characteristics, we extracted the following: study design, intervention type and duration, main objectives, and detailed descriptions of intervention content. Data from each study was summarized and aggregated. Meta-analysis was deemed not possible because of the wide variation of outcome measures used within the included studies.

The Cochrane risk of bias (RoB) assessment was conducted using the Cochrane RoB 2 tool specifically designed for Randomized Controlled Trials (RCTs) [33]. The assessment of each domain involved categorizing it as having “low RoB,” “some concerns,” or “high RoB.” After individual domain assessments, studies with only one out of five risk domains receiving a “some concerns” judgment were classified as having “low RoB.” Studies with two or more “some concerns” judgments were designated as having an overall assessment of “some concerns.” Studies with one domain assessed as “high RoB” were categorized as having an overall assessment of “high RoB.” The risk of bias (RoB) for each study was evaluated by two authors, with any discrepancies resolved through negotiation.

## Results

### Study selection

**Fig. 1:**
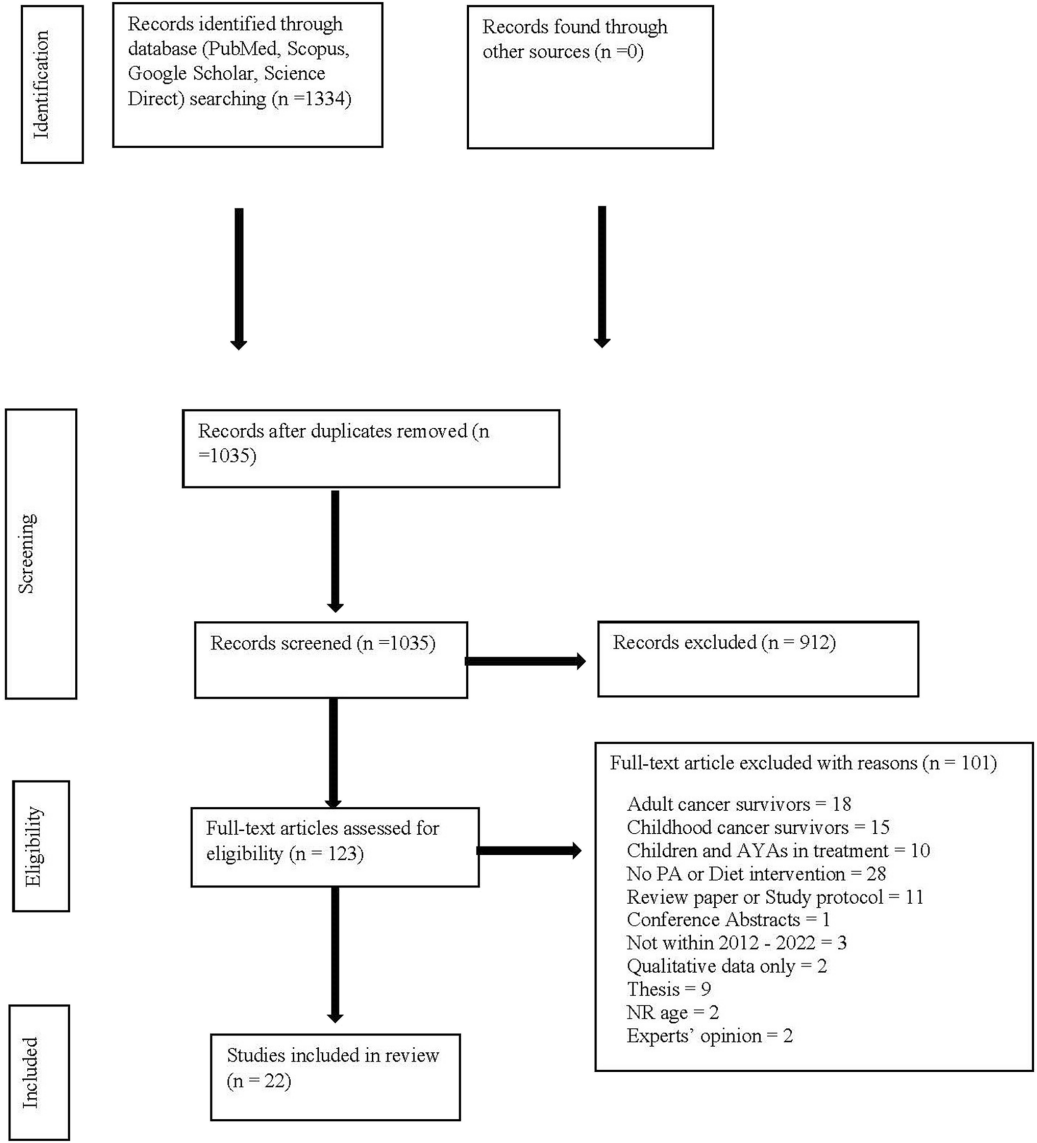
Flow diagram of the screening process

Figure 1 for the PRISMA flow diagram of the study selection process. The search strategy initially identified 1334 records, and 22 were included in the final review.

Table 1 for an overview of the study characteristics of included studies and Table 2 for characteristics of intervention types and outcomes. Most studies (20/22) were published between 2015 and 2022, with one study published in 2014 and one in 2013. The sample size ranged from 7 to 280 participants. Typically, participants were between 30 and 34 years of age. However, there was one study that recruited adolescent cancer survivors younger than 18 years of age. The three most common cancer diagnoses were lymphomas 14%, breast cancer 13%, and leukemia 10%. One study specifically recruited hematopoietic cell transplantation (HCT) survivors. A summary of the distribution of cancer types from the articles included in this review can be accessed in Table 1. Thirteen studies were randomized controlled trials, five were pre-post studies, one prospective descriptive single cohort study, one observational prospective two-group parallel study, one longitudinal prospective study, and one mixed-method study.

**Table 1:** Overview of included studies

*Characteristics*	*Categories*	*Number of studies*
Country	USA	13
	Norway	2
	Canada	4
	Australia	2
	Germany	1
Year of publication	2013	1
	2014	1
	2015	2
	2016	2
	2017	3
	2019	2
	2021	8
	2022	3
Sample size	≤ 25	9
	26–50	6
	51–75	1
	76–100	4
	≥ 101	2
Age	< 18	1
	18–24	3
	25–29	1
	30–34	13
	35–39	4
Cancer type	Breast cancer	12
	Lymphomas	11
	Leukemia	9
	Testes cancer	8
	Head and neck cancers	8
	Gynecological	7
	Colorectal cancer	5
	Gastric cancer	5
	Hematologic malignancies	4
	Thyroid cancer	3
	Ovarian cancer	3
	Sarcoma	2
	Solid tumors	2
	Soft tissue sarcoma	2
	Rhabdomyosarcoma	2
	Biphasic peritoneal mesothelioma	2
	Osteosarcoma	2

**Table 2:** Study characteristics, physical activity, and diet interventions of included studies

Author, year	Sample size (*n*)	Mean age (*y*)	Intervention typology	Weeks (*n*)	Time	Intensity (%)	Outcomes	Assessment tools
Valle et al. [34]	280	33.40	Mobile Health PA	24	-	-	PA min/wk MVPA Light PA Steps sedentary behavior	PA: ActiGraph GT3X + , participants wore it on their wrist for 7 days. Self-reported PA: GLTEQ. SBQ
Price and Brunet [35]	7	33.9	PA, Fruit Vegetable Consumption Teleconferencing	12	1 h/w face to face via teleconferencing	-	PA FVC Motivational processes	Self-reported: PA, FVC. Assessment of autonomy, competence, and relatedness in PA contexts: Exercise Treatment Self-Regulation Q. Dietary Self-Regulation Q. Interview
Johnson et al. [36]	49	33.6 ± 4.9	Fitbit and Facebook PA	12	Weekly texts or phone calls ≤ 5 min. ActiGraphs were worn for 7 days and then were returned	-	Daily minutes of subjective MVPA, light PA and ST, SDT constructs, and HQOL	MVPA, light-intensity PA, and ST: GT3X + ActiGraph SDT constructs: Behavioral Regulation in Exercise Q-2. QOL: Fatigue Symptom Inventory
Atkinson et al. [37]	43	20 ± 3	A^a^–RT^b^	10	2 sessions/w	A^a^: 50–85% HR max	Cardiorespiratory fitness, fatigue, QOL	Cardiorespiratory fitness: VO_2peak_ fatigue: FACIT-F, QOL: PEDS QL
Salchow et al. [38]	89	24.1 $\pm$ 6.3	Structured counseling-based PA	12	1 h of semi-structured interviews	-	Rates of participation in vigorous PA, amount, and intensity of the participants’ PA behavior, QOL	Rates of vigorous PA, amount and intensity of the participants’ PA behavior: IPAQ. QOL: EORTC and QLQ-C30
Price and Brunet [39]	16	33.9	PA, Fruit Vegetable Consumption Teleconferencing	12	1 h/w face to face via teleconferencing	-	Feasibility, PA, fruit vegetable consumption	Feasibility: recruitment, enrollment, adherence, retention, and attrition rates. PA participation: IPAQ–Short Form. BRFSS
Keadle et al. [40]	25 PA 26 PA + charity	36.8	eHealth PA with charity incentives	12	Weekly Newsletter email	-	Feasibility and increased PA	PA: ActivPAL (a research-grade device) was used for the calculation of daily steps. SR: physical function, fatigue, depression, and sleep disturbance. Motivational factors: BREQ-3
Miller et al. [41]	16	19.7	PA^c^	2	7 days at baseline, 2 weeks after the first two exercise sessions, and 2 weeks after the last two exercise sessions: Actigraph	-	MVPA, autonomy, competence, and relatedness, exercise motivation and fatigue	MVPA: Actigraph GT3x. Autonomy, competence, and relatedness: PNSE. Exercise motivation: BREQ Fatigue: FSA
Wurz et al. [42]	16	35.2 ± 5.6	PA^d^	12	4 weekly sessions of 25–45 min	A^a^: 40–75% HR	Feasibility EF task performance	Feasibility: number of participants, adherence to fMRI scans, outliers, missing data. EF: Letter n-back task and GO/NO GO task
De Nysschen et al. (2021)	24	16.6 ± 2.8	Exercise and Nutrition Education	8	Weekly 1 h PA	-	Health-related fitness: cardiovascular endurance, flexibility, muscular strength, endurance, anthropometrics. QoL, fatigue, PA self-efficacy, and nutrition education sessions with a dietician to enhance nutrition knowledge and dietary habit	Health-related fitness: wrist-based activity trackers (Fitbit Flex). QoL-fatigue: FACIT, QoL-PedsQL. PA self-efficacy: Godin Leisure-Time Q Nutrition knowledge and dietary habit: a 20-question nutrition quiz and 3-day dietary intake records were completed at baseline and post-intervention
Ketterl et al. [43]	20	18–39	RT^b^	12	3 in-person exercise sessions with an exercise physiologist and a progressive increase in individual RT^b^	-	Body composition metabolic fitness, strength testing, feasibility	Body composition: LBM and PFM. Strength and endurance assessments feasibility: number of enrolled participants, weekly follow-up phone calls with participants, number of participants who completed the intervention
Smith et al. [44]	35	21.1 ± 2.6	Rehabilitation Program	8	Weekly 1 h PA and 1 h education/self-management session	-	Feasibility, acceptability: adherence Program Ratings: exercise and PA, eating a healthy diet, accessing support in the community. Physical functioning, HQOL, Self-perception of performance and satisfaction	Physical Function measures PedsQL COPM Self-reported: health literacy, program evaluation
Wurz and Brunet [45]	16	32.8 ± 7.9	PA	12	2 weekly sessions^a^ unsupervised-week 3–4 = 20 min, week 5–6 = 25 min, week 7–8 = 30 min, week 9–12 = 30 min 2 weekly sessions^b^ supervised-week 1–6^f^ = 15–20 min, unsupervised-week 7–12 = 15–20 min	A^a^: 40–60 %HRR (week 1–8), 60–75 %HRR (week 9–12) RT^b^: 1–2 sets, 8–12 RM (week 1–6), 2–3 sets, 6–10 RM (week 7–12)	Feasibility, acceptability and adverse events, PA, psychological outcomes, participants encountered some difficulties when attempting to integrate PA into their daily routines	PA: GLTEQ Actigraph wGT3XP-BT Physical outcomes: Portable HR-200 height rod, Tanita TBF-310 GS scale, 30-s sit-to-stand test, HealthSmart Digital Blood Pressure Monitor, 6MWT Self-reported: Exercise Self-Efficacy scale, PSDQ-S, Rosenberg Global Self-Esteem Scale Qualitative Interviews
Lynch et al. [46]	46	37	Exercise, Nutritional/Behavioral Strategies and Health Lessons	52	3 d/w (women)/ 4 d/w (men): weight training 2 d/w: cardiovascular/ interval training 1 d/w: active recovery 1 d/w: rest day every 2 weeks: participants were given a new healthy habit to practice Each day: read lessons Biweekly emails: SR Any changes in upper or lower body symptoms	-	Feasibility, adherence, efficacy, and safety	Anthropometric measures: Stadiometer, Seca 876 scale, non-elastic measuring tape, Lange caliper Model EQ0014921. Feasibility: number of enrolled participants. Retention: number of participants attending 6/12-month study visit/number of enrolled SR. Adherence: average of mean percent Adherence to each of the 3 intervention components. Efficacy: changes in initial weight (percent weight loss). Safety: Number and type of adverse events
Rabin [47]	35	33.6 ± 4.0	Home-based PA and Meditation	12	Weekly engagement	-	Participants reporting PA barriers: schedule-related barriers, health-related barriers, other type of barrier	Intervention calls, PAR
Valle and Tate [48]	86	31.75	Facebook PA	12	Weekly engagement	-	Facebook engagement Differences in engagement by group format Types of Facebook posts The relationship between Facebook engagement and PA outcomes	Pedometer, BMI, PA: GLTEQ. Facebook engagement: Response posts, Likes, Participant-initiated posts, Interactions (sum of response posts, initiated posts, likes) SR: Facebook Intensity Scale, Fb engagement questions, perceptions of Fb group discussions, Perceptions of Facebook, Type of engagement: posts, likes and SR data
Gill et al. [49]	116	32.4 ± 6.5	Outdoor adventure therapy-PA	1	5–7 h per day/ 7 days: group and individualized instruction from professional guides and program staff	-	Comparison of the effects of an outdoor adventure therapy camp experience compared with a wait list control on PA effects on sedentary behavior, PA variety, self-efficacy, environmental change self-efficacy, perceived barriers to exercise, and PA enjoyment	Self-reported: First Descents physical exam and participant application forms, demographics survey PA: PAR, Sedentary behavior: two questions pertaining to the number of hours spent watching television and the number of hours spent seated in the previous 7 days and Sallis Self-Efficacy and Exercise Habits Survey, Environmental-Change Self-Efficacy, Sallis Barriers to PA Scale, Preferred Activities Q, Enjoyment of PA Q, Enjoyment of Inactive Recreation scale
Rabin et al. [50]	35	33.6 ± 4.0	Home-based PA and Meditation	12	Weekly engagement	-	Feasibility, acceptability. Weekly minutes of moderate-intensity PA, cardiovascular fitness, enhanced fitness, and mood	Feasibility: retention, intervention calls. Acceptability: Satisfaction rating. PA: PAR. Treadmill walk: One-mile walk test. Assessment of mood: POMS
Hauken et al. [51]	20	31.1	Rehabilitation Program	24	3 weeks of residential rehabilitation, with 1-week follow-up visits after 3 and 6 months	-	HRQOL, Physical capacities	HRQOL: EORTC QOL C-30. Physical capacities: physical fitness, lung capacity, and strength and body shape. Aerobic fitness test: Aastrand 6-min Cycle Test
[52]	86 41 (control)	30.8 IG	PA	12	Weekly engagement	-	MVPA levels to $\ge$ 150 min per week	PA: GLTEQ. Self-efficacy for PA: Self-Efficacy and Exercise Habits Survey. Social support for exercise: Adapted items from the Social Support and Exercise Survey
Hauken et al. [53]	16	30.8	Rehabilitation PA program	24	Weekly engagement	-	PA, participation in the program. Qualitative descriptions of participants	Participation: COMP. PA: condition, strength, and lung capacity
Valle et al. [54]	86 41 (control)	30.8	Facebook-based PA	12	Weekly engagement	-	MVPA, feasibility, QoL	PA: GLTEQ. QoL: FACT-G. Adherence and acceptability

*PA* physical activity, *MVPA* moderate-to-vigorous PA, *SBQ* Sedentary Behavior Questionnaire, *Q* questionnaire, *ST* sedentary time, *SDT* self-determination theory construct measures, *HQOL* health-related quality of life, *%HR max* percentage of heart rate maximum, *FACIT–F* Functional Assessment of Chronic Illness Therapy-Fatigue, *PEDS QL* Pediatric Quality of Life Inventory, *IPAQ* International Physical Activity Questionnaire, *EORTC QLQ-C30* European Organization for Research and Treatment of Cancer Quality of Life Questionnaire Core 30, *IPAQ* International Physical Activity Questionnaire—short form, *BRFSS* Behavioral Risk Factor Surveillance System–Fruit and Vegetable Questionnaire, *BREQ-3* behavioral regulation to exercise, *PNSE* psychological need satisfaction in exercise, *FCA* fatigue scale-adolescent, *NA* neural activity, *BMI* body mass index, *FACIT-F* fatigue and FACT-G v4, QoL concerns related to fatigue and well-being in cancer patients, *QoL-PedsQL* QoL pediatric–multidimensional fatigue teen and young adult report, *LBM* lean body mass, *PFM* percentage fat mass, *EORTC* Quality of Life Questionnaire C30, *GLTEQ* Godin Leisure-Time Exercise Questionnaire, *FACT-G* Functional Assessment of Cancer Therapy-General, *POMS* profile of mood states, *COMP* Canadian Occupational Performance Measure, *PAR* 7 day physical activity recall

^a^Aerobic Training

^b^Resistance Training

^c^A green exercise program comparing indoor and outdoor PA

^d^Neuroimaging with Executive functioning (EF) tasks to evaluate neural activity changes after PA (aerobic and strength training)

^e^Grip strength, 30-s chair stand assessment, one repetition maximum chest press, one repetition maximum leg press, and a 6-min walk test

Most studies included in this review sought to explore the feasibility and acceptability of physical activity (PA) interventions in adolescent and young adult cancer survivors (AYAs). Dietary interventions were combined with PA interventions; however, none of the included studies involved exclusively diet as an intervention. As a secondary objective, studies examined the effects of PA as well as diet interventions on health-related outcomes such as quality of life, fatigue, physical and mental health, and physical functioning.

### Risk of bias assessment

**Fig. 2:**
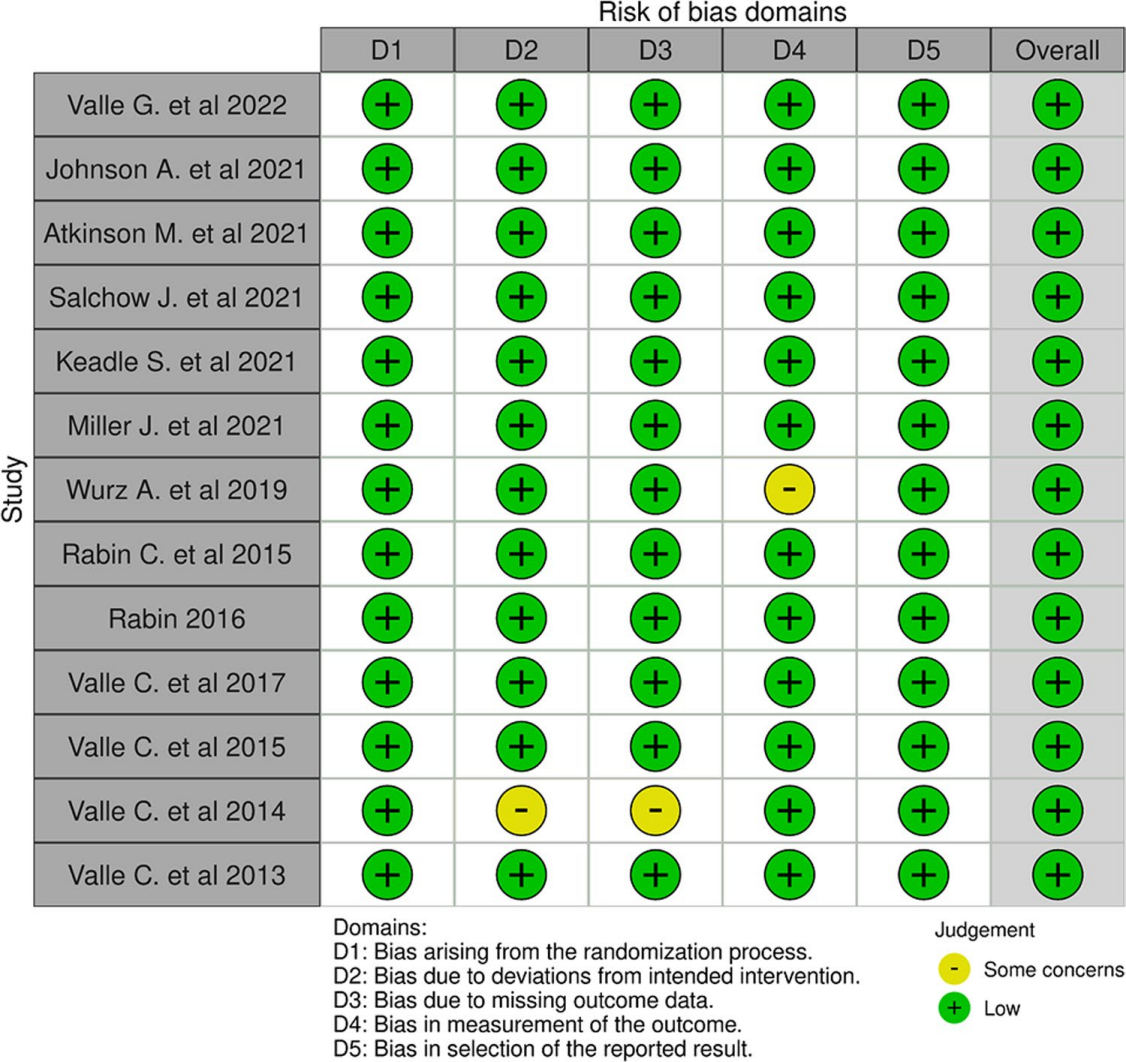
Risk of bias assessment summary

RoB evaluations for every study are summarized in Fig. 2. Overall, the judgments exhibited a “low” risk of bias. The designation of “some concerns” was assigned only to two studies [54],Wurz et al. 2019).

### PA and diet interventions

Physical activity (PA) interventions were mainly aerobic and resistance training and were individualized interventions. A high number of PA and diet interventions occurred in-person or in combination with online sessions, some studies involved exclusively online PA sessions too. In designing PA interventions, some studies employed health-behavior theories [52, 54],Lynch et al., 2016; [48] such as Social Cognitive Theory, while others [35, 36, 39] used a theoretical framework based on self-determination theory.

#### Diet interventions

A small minority of these studies comprised both diet and PA interventions. To explore both the feasibility as well as the motivational processes underlying Fruit and Vegetable consumption (FV) and physical activity, Price and Brunet designed a 12-week telehealth behavior change intervention for rural-living young adult cancer survivors [35, 39]. Participants met once a week with a health coach via teleconferencing,in both studies, a semi-structured interview took place post-intervention. Participants self-reported higher engagement with an increase in minutes of moderate-to-vigorous intensity PA (MVPA) per week post-intervention than pre-intervention and higher FV consumption per day post-intervention than pre-intervention [39]. In the sub-study, qualitative analysis demonstrated that the intervention enabled autonomous motivation and behavioral persistence in this population of young cancer survivors [35]. Similarly to these studies, Lynch et al. [46] tested the feasibility, efficacy, and safety of a 1-year long web-based weight loss intervention in young adult cancer survivors. The intervention compromised an exercise component, a healthy component with an introduction to dietary and lifestyle changes, and health lessons with various topics such as how to follow a healthy habit [46]. Upon completion of this web-based weight loss intervention, a 5.3% clinically significant loss in body weight was observed at 12 months [46]. Following the same combination of PA and Diet interventions, but with a different format, DeNysschen et al. [11] explored an 8-week in-person exercise and nutrition education program for AYA cancer survivors. With the goal of enhancing various areas of health-related fitness (i.e., cardiovascular endurance), wrist-based activity trackers were used, and QoL, fatigue, and physical activity self-efficacy were assessed at baseline and post-intervention too. Nutrition education sessions were also led by a Registered Dietician (RD) [11]. Significant improvement in fatigue, QoL, and areas of health-related fitness were observed, and additionally, significant differences in nutritional knowledge were observed post-intervention, but not in dietary intake [11].

#### PA interventions and rehabilitation

Drawing from rehabilitation research, Smith et al. [44] designed an 8-week, group-based, goal-oriented rehabilitation program (ReActivate) and tested its feasibility and its effect on physical and mental health, physical functioning, and occupational performance in AYA cancer survivors. The program consisted of 1 h of supervised PA per week followed by 1 h of education or self-management sessions and peer support. The results revealed a significant increase in physical functioning, health-related quality of life, program evaluation, and self-perception of performance and satisfaction. Similarly, Hauken et al. developed a complex rehabilitation program that involved 3 weeks of residential rehabilitation with 1-week follow-up visits after 3 and 6 months to examine the health-related quality of life (HRQOL) and physical capacities of YACS [51, 53]. During the PA intervention, the individual workout sessions were supervised by a physiotherapist, whereas the strength based-sessions, physical fitness, coordination, body awareness, and relaxation took place in a group [51]. Equally as Smith et al., health-related quality of life revealed a significant increase in overall HRQOL post-intervention. In addition, functional dimensions were also significantly better and fatigue significantly decreased [51]. Hauken et al. [53], with the use of the same rehabilitation program, explored whether this intervention would increase AYA cancer survivors’ participation and documented their experiences and thoughts regarding this goal-oriented experience. Participation did increase when considering rehabilitation programs for young cancer survivors, and coping and control were believed to be an important factor during the goal-oriented process [53].

#### Social media networking sites (SNS)-based and eHealth PA interventions

Valle et al. (2023) proposed the IMproving Physical Activity after Cancer Treatment (IMPACT) trial, where they assessed a mobile 6-month PA intervention on total PA minutes/week as well as MVPA, light PA, steps, and sedentary (ST) behaviors in YACS. The difference between the intervention group (IMPACT) and the self-help group was that the former along with a series of digital tools and an individual video session additionally received a 6-month program that included behavioral lessons, Facebook prompts, tailored feedback and text messages, and adaptive goal setting (Valle et al., 2023). The intervention did not produce significant differences in total, between PA groups; however, an increase in MVPA in favor of the IMPACT group was detected (Valle et al., 2023). Another Facebook-related PA intervention was developed by Johnson et al. [36], with the aim of increasing daily steps. Researchers created a 12-week Fitbit Flex and Facebook study,the intervention group received a PA tracker (Fitbit Flex), weekly individualized goal setting (conducted by text (primarily) or phone), access to a Facebook group for reflection and discussion, the option to choose a PA “buddy,” and finally text messages to fulfill basic psychological needs [36]. Findings revealed a significant reduction in mean ST behavior from baseline to follow-up between the two groups. Moreover, participants who were “very engaged” on Facebook had an average lower minutes per day of ST in comparison with the “not engaged” ones [36]. Staying on social media networking sites (SNS)-based PA interventions, Valle et al. designed a 12-week Facebook (FITNET) intervention to enhance levels of MVPA in YACS and investigated whether changes in social cognitive theory constructs (self-efficacy, social support, and self-monitoring) will act as mediators between the intervention and the changes in PA [48, 52, 54]. An increase in MVPA was found albeit non-significant for the FITNET group, and social support from friends and self-monitoring and changes in moderate-to-vigorous PA were positively associated for both groups [52, 54]. Taking into consideration the observed high level of Facebook engagement, with over 84% of participants engaged to some degree, and the lower levels of ST behavior in Johnson et al., along with the potential increases in MVPA in Valle et al., reveal that social media-based PA intervention can potentially facilitate improvements in health behavior and change among AYA cancer survivors.

Similarly to Valle et al., another study that was included used Fitbit and created an eHealth intervention with charity-based incentives to raise PA levels among young adult cancer survivors [40]. Both groups (PA alone and PA + Charity) had to accomplish a specified number of daily steps,the PA + Charity group could additionally earn money to spend on charities of their choosing every time they reached their step goal [40]. The PA + Charity group was significantly more motivated to exercise, more satisfied with the program, and demonstrated a significant increase in daily steps in comparison to the PA-alone group [40].

#### Outdoor and indoor PA interventions

A Green Exercise program was developed by Miller et al. [41] to compare physical activity and fatigue levels throughout indoor and outdoor exercise sessions in AYA cancer survivors, and this was a RCT crossover group trial,one group finished first with the indoor sessions and then started the outdoor sessions, whereas the other group started with outdoor followed by indoor sessions. Findings revealed a significantly higher intensity in exercise during outdoor sessions compared to indoor sessions among AYAs. As for fatigue, exercise motivation, and physical activity measures, there were no statistically significant differences in the weeks following indoor exercises in comparison with outdoor sessions [41]. Outdoor activities were also used in Grill et al. (2016) during a 7-day adventure therapy camp to improve PA levels in young adult cancer survivors; sedentary behavior and PA correlates such as PA variety, exercise self-efficacy, and perceived barriers to exercise were used. After 1 week and during the 3-month follow-up, the camp group demonstrated a significant increase in physical activity levels compared to controls; they also spent significantly less time sitting and watching television when measured after 1 week but not at follow-up (Grill et al., 2016). Considering PA correlates, a significant decrease in perceived barriers was spotted, and for instance, a small but significant reduction in excuses was revealed for the camp group after 1 week but once again not at follow up (Grill et al., 2016).

#### Resistance training for hematopoietic cell transplant survivors (HCT)

A 12-week combined in-person and remote study by Ketterl et al. [43] explored a resistance training (RT) intervention for hematopoietic cell transplant survivors (HCT) during the early post-HCT period. This intervention primarily aimed at enhancing lean body mass (LBM) deficits and metabolic fitness in HTC survivors,a progressive program where participants had to exercise for 2 to 3 days per week [43]. Findings indicated that LBM was either stable or increased at 12 weeks, and at the 1-year follow-up, participants also reported that the intervention was enjoyable and that they would recommend it to a friend or an HCT survivor [43].

#### Other PA interventions

A population of AYA cancer survivors who have recently completed their cancer treatment participated in a 10-week exercise program consisting of progressive aerobic and resistance exercise with the aim of enhancing their cardiorespiratory fitness (VO2_peak_), levels of fatigue, and quality of life [37]. There was indeed a significant enhancement in VO2_peak_ at 10 weeks for the intervention group compared to controls, but this did not continue at 6 months follow-up thus fatigue and quality of life did not present significant changes between groups [37]. Researchers inferred that this exercise program can potentially accelerate improvement in cardiorespiratory fitness following cancer treatment,however, to sustain this effect, a maintenance program might be helpful [37]. Moving on to another PA intervention, Salchow et al. [38] examined the effect of a counseling-based PA program focusing on improving vigorous physical activity behavior among AYAs. Sixty-minute individualized PA interviews were conducted (intervention group), and the primary goal was defined as $\ge$ 9 MET hours per week of vigorous activity [38]. The results did not indicate a significant difference between the intervention and the control group on $\ge$ 9 MET hours per week of vigorous activity; hence, the researchers concluded that this counseling-based PA program did not have a significant impact on PA levels among AYAs [38].

Furthermore, Wurz et al. (2019) investigated the impact of a 12-week PA intervention with four weekly PA sessions focusing on aerobic and strength training; the aerobic training was unsupervised, whereas the strength training was supervised by a certified exercise physiologist during the first 6 weeks and unsupervised for the final 6 weeks. Participants were faced with challenges in integrating PA into their daily lives for instance, due to busy schedules or difficulties in planning. Furthermore, the transition from supervised to unsupervised training was deemed difficult for them (Wurz et al., 2019). Rabin [47] also explored potential barriers that survivors come to face when considering daily exercise. They inferred that 27% of barriers are categorized as either schedule-related, health-related, psychological, or weather-related barriers among AYA survivors. As a result, PA interventions should be adjusted in overcoming challenges if they are to improve physical activity in this targeted population (Wurz et al., 2019). For instance, Rabin et al. [50] combined exercise with meditation and found a significant increase in PA post-treatment for the intervention group, along with enhanced fitness levels and better mood.

#### Executive functioning tasks and PA intervention

Wurz et al. [42] conducted a proof-of-concept sub-study and explored executive functioning tasks (EF) and neural activity changes upon completion of a PA intervention in AYA cancer survivors. Their findings suggest that PA may potentially lead to better neural activity in areas of the brain that are responsible for working memory, planning complex movements, cognitive control, motor control, response inhibition, and decision making [42]. This is the only study included in this review that paired neural activity and PA activity among AYA cancer survivors as well as their suggested implications for this population.

### Feasibility and acceptability of PA and Diet interventions

Overall, studies included in this systematic review demonstrated that PA and diet interventions were both feasible and acceptable among adolescent and young adult cancer survivors. The high retention rates of each study showed that most participants accepted the interventions. For example, the retention rate was 59% at 6 and 49% at 12 months in Lynch et al. [46], 94% in Wurz et al. (2019), and 77% in Rabin et al. [50]. Moreover, there was only a 10% attrition for the primary outcome assessment at 10 weeks in Atkinson et al. [37], and 89% of participants in Ketterl et al. [43] completed the weekly coaching phone calls and reportedly did a median of two exercise sessions per week. In terms of satisfaction, participants reported that they would recommend this RT intervention to a friend or a HCT survivor and shared that they enjoyed the intervention [43]. Additionally, the eHealth intervention by Keadle et al. [40] was acceptable and was deemed satisfying by both groups with the PA + Charity group being sig. more satisfied with the program compared to PA alone. In Price and Brunet [39], the subjects believed that the duration of the intervention was sufficient for developing habits and routines for PA and FV consumption. Certain barriers were also reported by participants that can potentially hinder their daily physical activity. Rabin [47] identified the following barriers: competing family or work demands (62.5%), a general lack of time (50%), health issues (43.8%), and pain (28.1%). Grill et al. (2016) found that after a 7-day adventure therapy camp experience, the perceived barriers presented a significant decrease for the intervention group and participants made significantly less excuses, and still, this effect did not persist at the 3-month assessment. Considering goal setting, AYAs reported difficulty in setting realistic and specific PA goals and prioritizing them. Nevertheless, the authors reported that the structure of the rehabilitation program facilitated and helped participants during this process [53]. In Wurz et al. (2019), participants felt that the transition from supervised to unsupervised training sessions was challenging. Hence, taking into careful consideration the specific methods used in these interventions is ultimately a necessary step before they are deemed feasible and acceptable (Wurz et al., 2019).

## Discussion

The aim of the present review was to study in a systematic way the interventions around PA and diet and their effectiveness for AYAs cancer survivors, to reveal the knowledge regarding the essence of health behaviors such as PA and diet in this distinct subgroup among oncology patients, and also to critically evaluate the existing literature, the gaps, and its limitations. For this reason, a systematic review of the existing literature was carried out seeking data to identify and map PA and diet and nutrition interventions in the target group of AYA cancer survivors and secondarily to examine their qualitative characteristics. The studies included in this review focused mainly on examining the feasibility and acceptability of physical activity (PA) interventions for adolescent and young adult (AYA) cancer survivors while also exploring the potential impact of these interventions on health-related outcomes as a secondary objective.

Regarding the first aim, we identified studies that examined a type of PA and/or diet intervention among AYA cancer survivors. Considering PA interventions, the studies exhibit a high degree of heterogeneity which is consistent with the findings of the earlier systematic review by Munsie et al. [15] and the scoping review of Caru et al. [55]. These reviews noted that PA intervention studies conducted among AYA cancer survivors exhibit a wide range of variations in factors such as study design, types of PA interventions used, and primary goals. This lack of uniformity makes it difficult to make direct comparisons among these studies. Nonetheless, drawing from the included studies certain patterns did arise. For instance, in terms of the modality of the interventions, most of the studies included in this review used at least one form of digitally based PA interventions (Facebook-based, teleconferencing, mobile app, Fitbit trackers, mHealth intervention). One advantage in the use of digitally based interventions is that they provide an accessible and easy way of applying health behavior change, particularly, among rural-living young cancer survivors [35, 39]. Furthermore, when social media networking sites (SNS)-based PA interventions were combined with charity-based incentives, greater physical activity was observed in AYA cancer survivors [40]. Valle et al. (2017) additionally inferred that by examining the ways in which AYAs interact with each other during a SNS-based PA intervention can inform us about the shared experiences that these young people face and facilitate improvements in health behavior change. These types of interventions might be designed to provide information to prompt adherence to intervention, to connect AYA survivors with peer support, and to replace face-to-face counseling. Their role widely varies, therefore deciding beforehand the targeted research areas is a crucial step in the design of digital health interventions [56]. When evaluating outdoor and indoor PA sessions, Grill et al. (2016) demonstrated that after spending 5 to 7 h in an outdoor adventure activity, there was a significant reduction in sedentary behavior and an increase in PA levels for the camp group. Analogously, Miller et al. [41] revealed a higher PA intensity when AYAs exercised outdoors compared to indoors. Another common theme was that nearly half of the included studies consisted of follow-up assessments, as was signified in Hauken et al. [51], consistent follow-up assessments are crucial in rehabilitation programs after cancer treatment. In adopting a follow-up assessment, researchers can observe whether the early positive effects continued in the long-run and were adopted by AYA cancer survivors as a more permanent healthy way of living. Social support was found to be potentially linked to enhanced PA outcomes. Drawing from Social Cognitive Theory (Badura, 1986), Valle et al. [54] found positive associations for both groups (FITNET and self-help group) between social support from friends and self-monitoring and changes in MVPA. Likewise, as participants in a telehealth intervention built their confidence levels, feelings of connection and engagement in physical activity with others appeared [35]. Henceforth, competence is related to feelings of sharing the same goal. The gaps in the existing literature are even greater regarding diet interventions for this specific group of cancer survivors. More specifically, only few studies used in this systematic review examined nutrition or diet among AYA cancer survivors [46],De Nysschen et al., 2021; [35, 39], with nutrition being formally part of the overall intervention. However, their results were quite promising showing a significant weight loss post-intervention [46, 54] and significant differences in nutrition knowledge through healthy choices, a better understanding of nutrition labels, and detecting of saturated fat (De Nysschen et al., 2021). Similarly with the previous results, Campbell et al. [57] also demonstrated the efficacy of a lifestyle intervention based on the Diabetes Prevention Program and found an average weight loss of 3.8 ± 5.0 kg and a decrease in body mass index, percent body fat, and waist and hip circumferences at 24 weeks among early-stage breast cancer survivors. Reflecting on these findings, evidence shows that childhood cancer survivors experience excessive weight gain early in treatment and risk developing cardiovascular diseases,hence, Zhang, Kelly, and Must [58] emphasized the importance of healthy lifestyle choices starting as early as cancer treatment begins, to try and diminish these risks.

Included studies in this systematic review examined potential barriers that AYA cancer survivors come to face in their effort to increase PA activity. For example, Grill et al. (2016) observed a significant decrease in perceived barriers (i.e., excuses) for the intervention group compared to controls; nonetheless, this slight reduction did not emerge when conducting the follow-up assessment. Wurz et al. (2019) also discovered that the participants’ busy schedules or difficulties in planning were seen as obstacles in the integration of PA into daily living. Similarly, Rabin [47] who evaluated barriers to exercise experienced by young adult cancer survivors inferred that 27% of barriers were schedule-related, health-related, psychological as well as weather-related barriers. Previous studies such as Wu et al. [59] have found that facilitators of exercise and healthy eating included social relationships or cognitive motivators, whereas factors that hindered participation were lack of resources, negative thoughts and feelings, and negative social and environmental influences. They also underlined the importance of taking into consideration the desire for social support when developing interventions for young cancer survivors [59]. Having been diagnosed with cancer as a young individual (adolescent and young adult) can potentially create a distance from other people when one must deal with a series of challenging events from diagnosis to treatment and then to rehabilitation, and for this reason, social support can justifiably facilitate better health outcomes during PA and Diet interventions.

One of the strengths of this systematic review was the inclusion of high-quality research articles (*n* = 13 RCTs) which provided the opportunity for an in-depth qualitative analysis of findings and the emergence of helpful themes in PA and diet interventions among AYA cancer survivors. As far as we know, this is also the first review that systematically identified and synthesized the most current and updated PA and diet interventions for this target group within the chosen time range. At the same time, due to the heterogeneity between the characteristics of the interventions included in this review, a meta-analysis was deemed not possible, and consequently, a statistical synthesis of the included findings was not conducted. Another limitation of this review is the specific time range (2012–2022); it is possible that by having an unlimited time range scientifically interesting patterns might have arisen. Some limitations regarding these studies were the high number of female participants as opposed to male, the high number of certain types of cancer (i.e., breast cancer), the lack of control groups in certain studies, the lack of diversity in terms of location (the majority were conducted in the USA), and finally the small samples sizes.

## Conclusion

The current systematic review has shown the diverse spectrum of physical activity (PA) and diet interventions that have been conducted in adolescent and young adult cancer survivors (AYA) during the selected time frame. These were deemed feasible, acceptable, and well-adhered by AYAs. There are still large gaps in knowledge in the designing and implementation of these types of interventions. Higher recruitment and retention rates, bigger sample sizes with diverse ethnic groups, and various cancer diagnoses should be implemented in the design of these interventions. Social support and ways to overcome barriers faced by AYAs will enable more active participation in physical activity. The utmost goal is a formal PA and Diet intervention plan for AYA survivors, but to succeed in this, more research needs to be conducted to fill in the existing gaps.

### Supplementary Information

Below is the link to the electronic supplementary material.

Supplementary file1 (DOCX 29 KB)

## Data Availability

No datasets were generated or analysed during the current study.

## References

[1] Barr RD, Ferrari A, Ries L (2016). Cancer in adolescents and young adults: a narrative review of the current status and a view of the future. JAMA Pediatr.

[2] Arnett JJ (2000). Emerging adulthood. A theory of development from the late teens through the twenties. Am Psychol.

[3] Adams SC, Herman J, Lega IC, Mitchell L, Hodgson D, Edelstein K, Travis LB, Sabiston CM, Thavendiranathan P, Gupta AA (2021). Young adult cancer survivorship: recommendations for patient follow-up, exercise therapy, and research. JNCI Cancer Spectr..

[4] Chao C, Xu L, Bhatia S (2016). Cardiovascular disease risk profiles in survivors of adolescent and young adult (AYA) cancer: the Kaiser permanente AYA cancer survivors study. J Clin Oncol..

[5] Janssen SHM, van der Graaf WTA, van der Meer DJ, Manten-Horst E, Husson O (2021). Adolescent and young adult (AYA) cancer survivorship practices: an overview. Cancers.

[6] Jensen MV, Rugbjerg K, de Fine Licht S, Johansen C, Schmiegelow K, Andersen KK, Winther JF (2018). Endocrine late effects in survivors of cancer in adolescence and young adulthood: a Danish population-based cohort study. JAMA Netw Open.

[7] Rock, C. L., Thomson, C. A., Sullivan, K. R., Howe, C. L., Kushi, L. H., Caan, B. J., Neuhouser, M. L., Bandera, E. V., Wang, Y., Robien, K., Basen-Engquist, K. M., Brown, J. C., Courneya, K. S., Crane, T. E., Garcia, D. O., Grant, B. L., Hamilton, K. K., Hartman, S. J., Kenfield, S. A., Martinez, M. E., … McCullough, M. L. (2022). American Cancer Society nutrition and physical activity guideline for cancer survivors. CA Cancer J Clin, 72(3), 230–262. 10.3322/caac.21719. 10.3322/caac.21719 35294043

[8] Islami F, Goding Sauer A, Miller KD, Siegel RL, Fedewa SA, Jacobs EJ, McCullough ML, Patel AV, Ma J, Soerjomataram I, Flanders WD, Brawley OW, Gapstur SM, Jemal A (2018). Proportion and number of cancer cases and deaths attributable to potentially modifiable risk factors in the United States. CA Cancer J Clin.

[9] Li T, Wei S, Shi Y (2016). The dose-response effect of physical activity on cancer mortality: findings from 71 prospective cohort studies. Br J Sports Med.

[10] Pugh G, Gravestock HL, Hough RE, King WM, Wardle J, Fisher A (2016). Health behavior change interventions for teenage and young adult cancer survivors: a systematic review. J Adolesc Young Adult Oncol.

[11] DeNysschen CA, Panek-Shirley LM, Zimmerman B (2021). Exercise with nutrition education to improve quality of life of adolescent and young adult cancer survivors: a pilot study. J Adolesc Young Adult Oncol.

[12] Murnane A, Gough K, Thompson K, Holland L, Conyers R (2015). Adolescents and young adult cancer survivors: exercise habits, quality of life and physical activity preferences. Support Care Cancer.

[13] Cormie P, Zopf EM, Zhang X, Schmitz KH (2017). The impact of exercise on cancer mortality, recurrence, and treatment-related adverse effects. Epidemiol Rev.

[14] Van der Meer, D. J., Karim-Kos, H. E., van der Mark, M., Aben, K. K., Bijlsma, R. M., Rijneveld, A. W., ... & Husson, O. (2020). Incidence, survival, and mortality trends of cancers diagnosed in adolescents and young adults (15–39 years): a population-based study in The Netherlands 1990–2016. Cancers, 12(11), 3421. 10.3390/cancers12113421 PMC7698904 33218178

[15] Munsie C, Ebert J, Joske D, Ackland T (2019). The benefit of physical activity in adolescent and young adult cancer patients during and after treatment: a systematic review. J Adolesc Young Adult Oncol.

[16] Valle CG, Pinto BM, LaRose JG, Diamond M, Horrell LN, Nezami BT, Hatley KE, Coffman EM, Polzien K, Hales DP, Deal AM, Rini CM, Rosenstein DL, Tate DF (2021). Promoting physical activity in young adult cancer survivors using mHealth and adaptive tailored feedback strategies: design of the improving physical activity after cancer treatment (IMPACT) randomized controlled trial. Contemp Clin Trials.

[17] Mustian KM, Sprod LK, Janelsins M, Peppone LJ, Mohile S (2012). Exercise recommendations for cancer-related fatigue, cognitive impairment, sleep problems, depression, pain, anxiety, and physical dysfunction: a review. Oncol Hematol Rev.

[18] Spathis A, Hatcher H, Booth S, Gibson F, Stone P, Abbas L, Barclay M, Brimicombe J, Thiemann P, McCabe MG, Campsey R, Hooker L, Moss W, Robson J, Barclay S (2017). Cancer-related fatigue in adolescents and young adults after cancer treatment: persistent and poorly managed. J Adolesc Young Adult Oncol.

[19] Anderson E, Durstine JL (2019). Physical activity, exercise, and chronic diseases: a brief review. Sports Medicine and Health Science.

[20] Husson O, Zebrack BJ (2017). Perceived impact of cancer among adolescents and young adults: relationship with health-related quality of life and distress. Psychooncology.

[21] Saris LMH, Vlooswijk C, Kaal SEJ, Nuver J, Bijlsma RM, van der Hulle T, Kouwenhoven MCM, Tromp JM, Lalisang RI, Bos MEMM, van der Graaf WTA, Husson O (2022). A negative body image among adolescent and young adult (AYA) cancer survivors: results from the population-based SURVAYA study. Cancers.

[22] Vani MF, Sabiston CM, Santa Mina D, Trinh L (2022). “Your body is not at all where you left it”: adolescent and young adult cancer survivors’ experiences transitioning back into physical activity after treatment. Qual Health Res.

[23] Matarazzo JD, Hammonds BL, Scheirer CJ (1984). Behavioral immunogens and pathogens in health and illness. Psychology and Health.

[24] Skiba MB, McElfresh JJ, Howe CL, Crane TE, Kopp LM, Jacobs ET, Thomson CA (2020). Dietary interventions for adult survivors of adolescent and young adult cancers: a systematic review and narrative synthesis. J Adolesc Young Adult Oncol.

[25] Milliron BJ, Packel L, Dychtwald D, Klobodu C, Pontiggia L, Ogbogu O, Barksdale B, Deutsch J (2022). When eating becomes torturous: understanding nutrition-related cancer treatment side effects among individuals with cancer and their caregivers. Nutrients.

[26] Kohler LN, Garcia DO, Harris RB, Oren E, Roe DJ, Jacobs ET (2016). Adherence to diet and physical activity cancer prevention guidelines and cancer outcomes: a systematic review. Cancer Epidemiol Biomarkers Prev.

[27] Skiba MB, Jacobs ET, Crane TE, Kopp LM, Thomson CA (2022). Relationship between individual health beliefs and fruit and vegetable intake and physical activity among cancer survivors: results from the health information national trends survey. J Adolesc Young Adult Oncol.

[28] Warner EL, Nam GE, Zhang Y, McFadden M, Wright J, Spraker-Perlman H, Kinney AY, Oeffinger KC, Kirchhoff AC (2016). Health behaviors, quality of life, and psychosocial health among survivors of adolescent and young adult cancers. J Cancer Surviv.

[29] Hurtado-Barroso S, Trius-Soler M, Lamuela-Raventós RM, Zamora-Ros R (2020). Vegetable and fruit consumption and prognosis among cancer survivors: a systematic review and meta-analysis of cohort studies. Adv Nutr.

[30] Schwingshackl L, Schwedhelm C, Galbete C, Hoffmann G (2017). Adherence to Mediterranean diet and risk of cancer: an updated systematic review and meta-analysis. Nutrients.

[31] Berdan CA, Tangney CC, Scala C, Stolley M (2014). Childhood cancer survivors and adherence to the American Cancer Society Guidelines on Nutrition and Physical Activity. J Cancer Surviv.

[32] Moher  D, Liberati  A, Tetzlaff  J, Altman  DG, Group P (2009). Preferred reporting items for systematic reviews and meta-analyses: the PRISMA statement. PLoS Med.

[33] Akl E, Altman D, Aluko P, Askie L, Beaton D, Berlin J et al. Cochrane handbook for systematic reviews of interventions 2019

[34] Valle CG, Diamond MA, Heiling HM, Deal AM, Hales DP, Nezami BT, Pinto BM, LaRose JG, Rini CM, Tate DF (2022). Effect of an mHealth intervention on physical activity outcomes among young adult cancer survivors: the IMPACT randomized controlled trial. Cancer.

[35] Price J, Brunet J (2022). Understanding rural-living young adult cancer survivors’ motivation during a telehealth behavior change intervention within a single-arm feasibility trial. Health Informatics J.

[36] Johnson AM, K. Baker S, Haviland MJ, Syrjala KL, Abbey-Lambertz M, Chow EJ, Mendoza JA (2022). A pilot randomized controlled trial of a Fitbit- and Facebook-based physical activity intervention for young adult cancer survivors. J Adolesc Young Adult Oncol.

[37] Atkinson M, Murnane A, Goddard T (2021). A randomized controlled trial of a structured exercise intervention after the completion of acute cancer treatment in adolescents and young adults. Pediatr Blood Cancer.

[38] Salchow J, Koch B, Mann J, von Grundherr J, Elmers S, Dwinger S, Escherich G, Vettorazzi E, Reer R, Sinn M, Baumann F, Bokemeyer C, Stein A, Jensen W (2021). Effects of a structured counselling-based intervention to improve physical activity behaviour of adolescents and young adult cancer survivors-the randomized phase II Motivate AYA-MAYA trial. Clin Rehabil.

[39] Price J, Brunet J (2021). Feasibility and acceptability of a telehealth behavior change intervention for promoting physical activity and fruit and vegetable consumption among rural-living young adult cancer survivors. J Psychosoc Oncol.

[40] Keadle SK, Meuter L, Phelan S (2021). Charity-based incentives motivate young adult cancer survivors to increase physical activity: a pilot randomized clinical trial. J Behav Med.

[41] Miller JM, Sadak KT, Shahriar AA, Wilson NJ, Hampton M, Bhattacharya M, Towle A, Turcotte LM (2021). Cancer survivors exercise at higher intensity in outdoor settings: the GECCOS trial. Pediatr Blood Cancer.

[42] Wurz A, Ayson G, Smith AM, Brunet J (2021). A proof-of-concept sub-study exploring feasibility and preliminary evidence for the role of physical activity on neural activity during executive functioning tasks among young adults after cancer treatment. BMC Neurol.

[43] Ketterl TG, Ballard S, Bradford MC, Chow EJ, Jenssen K, Myers S, Rosenberg AR, Van Doren M, Baker KS (2021). Feasibility and acceptability of a home-based resistance training intervention in adolescent and young adult hematopoietic cell transplant survivors. Pediatr Blood Cancer.

[44] Smith AH, Murnane A, Thompson K, Mancuso S (2019). ReActivate—a goal-orientated rehabilitation program for adolescent and young adult cancer survivors. Rehabilitation Oncology.

[45] Wurz A, Brunet J (2019). Exploring the feasibility and acceptability of a mixed-methods pilot randomized controlled trial testing a 12-week physical activity intervention with adolescent and young adult cancer survivors. Pilot and Feasibility Studies.

[46] Lynch SM, Stricker CT, Brown JC, Berardi JM, Vaughn D, Domchek S, Filseth S, Branas A, Weiss-Trainor E, Schmitz KH, Sarwer DB (2017). Evaluation of a web-based weight loss intervention in overweight cancer survivors aged 50 years and younger. Obes Sci Pract.

[47] Rabin C (2017). Barriers to increasing physical activity among young adult cancer survivors. J Adolesc Young Adult Oncol.

[48] Valle CG, Tate DF (2017). Engagement of young adult cancer survivors within a Facebook-based physical activity intervention. Translational Behavioral Medicine.

[49] Gill E, Goldenberg M, Starnes H, Phelan S (2016). Outdoor adventure therapy to increase physical activity in young adult cancer survivors. J Psychosoc Oncol.

[50] Rabin C, Pinto B, Fava J (2016). Randomized trial of a physical activity and meditation intervention for young adult cancer survivors. J Adolesc Young Adult Oncol.

[51] Hauken MA, Holsen I, Fismen E, Larsen TM (2015). Working toward a good life as a cancer survivor: a longitudinal study on positive health outcomes of a rehabilitation program for young adult cancer survivors. Cancer Nurs.

[52] Valle CG, Tate DF, Mayer DK, Allicock M, Cai J (2015). Exploring mediators of physical activity in young adult cancer survivors: evidence from a randomized trial of a Facebook-based physical activity intervention. J Adolesc Young Adult Oncol.

[53] Hauken MA, Holsen I, Fismen E, Larsen TM (2014). Participating in life again: a mixed-method study on a goal-orientated rehabilitation program for young adult cancer survivors. Cancer Nurs.

[54] Valle CG, Tate DF, Mayer DK, Allicock M, Cai J (2013). A randomized trial of a Facebook-based physical activity intervention for young adult cancer survivors. J Cancer Surviv.

[55] Caru M, Levesque A, Rao P, Dandekar S, Terry C, Brown V, McGregor L, Schmitz K (2022). A scoping review to map the evidence of physical activity interventions in post-treatment adolescent and young adult cancer survivors. Crit Rev Oncol Hematol.

[56] Devine KA, Viola AS, Coups EJ, Wu YP (2018). Digital health interventions for adolescent and young adult cancer survivors. JCO Clinical Cancer Informatics.

[57] Campbell KL, Van Patten CL, Neil SE, Kirkham AA, Gotay CC, Gelmon KA, McKenzie DC (2012). Feasibility of a lifestyle intervention on body weight and serum biomarkers in breast cancer survivors with overweight and obesity. J Acad Nutr Diet.

[58] Zhang FF, Kelly MJ, Must A (2017). Early nutrition and physical activity interventions in childhood cancer survivors. Curr Obes Rep.

[59] Wu YP, Yi J, McClellan J, Kim J, Tian T, Grahmann B, Kirchhoff AC, Holton A, Wright J (2015). Barriers and facilitators of healthy diet and exercise among adolescent and young adult cancer survivors: implications for behavioral interventions. J Adolesc Young Adult Oncol.

