# Supplementary material for: Interventions about physical activity and diet and their impact on adolescent and young adult cancer survivors: a Prisma systematic review
Source: Support Care Cancer. 2024 May 13;32(6):342. doi: 10.1007/s00520-024-08516-0 (PMC11090968; doi:10.1007/s00520-024-08516-0)
Supplement: Supplementary file 1 — Supplementary file1 (DOCX 29 KB) [file 520_2024_8516_MOESM1_ESM.docx]

**Interventions about physical activity and diet and their impact on Adult and Young Adults cancer survivors: A Prisma systematic review**

Vasilopoulou M.,^1^ Asimakopoulou Z.,^1^ Velissari I.^1,2^, Viha A.^2^, Rizogianni M.^1^, Pusa S^3^, Stöven S.^13^, Ficarra S^4^., BiancoΑ.^4^, Jiménez-Pavón D.^5,6,7^, Ponce Gonzalez Jesus G.^6,8^, Tavares Paula C. ^9^, Gomes B.^9^, Bayer D.^10^, Silva S. ^11^, Nogueira C. ^12^, Papakonstantinou S.^14^, Musa K.^15^, Glorioso F.^16^ and A.Vantarakis*^1^

*Corresponding Author:

Apostolos Vantarakis, B.Sc., M.Sc., Ph.D., Prof.

Orcid- ID: 0000-0003-4411-3739

[avanta@upatras.gr](mailto:avanta@upatras.gr)

Department of Public Health, Medical School

University of Patras

University Campus, 26504 Rio Achaia

Tel. 302610969875

Patras, Greece

**Supplementary File:**

**Search Strategy for one database**

**Search Strategy for one database**

**Population**

Adolescent and young adult cancer survivors [‘adolescent’, ‘young adult’, ‘young cancer survivors’, ‘young adult cancer survivors’, ‘AYA cancer survivors’, ‘cancer’, ‘survivors’, ‘survivorship’].

**Interventions**

Exercise or Physical Activity and Diet Interventions [‘exercise’, ‘physical activity’, ‘diet’, ‘nutrition’, ‘health behaviours’, ‘lifestyle’, ‘intervention’].

**Comparators**

Usual care, wait-list control.

**Outcomes**

Cardiorespiratory Fitness, Strength, Fatigue, Quality of life.

**Study Design**

Randomized Controlled Trials, Mixed-Method studies, Pre-post studies, Prospective descriptive single cohort studies, Observational prospective two group parallel study, Longitudinal prospective study.

**Limitations**

Peer-reviewed papers, published between 2012 and 2022.

**Filters**

Publication date, Article, English

**Keywords**

1. adolescent
2. young adult
3. cancer survivors
4. health behaviours
5. physical activity
6. exercise
7. nutrition
8. diet
9. lifestyle
10. intervention

**Google Scholar**

1+2+3+4+5+6+7+8+9+10 = 329 results

The search was conducted up until 22^nd^ of February 2022.
